# The role of TGFBI in mesothelioma and breast cancer: association with tumor suppression

**DOI:** 10.1186/1471-2407-12-239

**Published:** 2012-06-13

**Authors:** Bingyan Li, Gengyun Wen, Yongliang Zhao, Jian Tong, Tom K Hei

**Affiliations:** 1 School of Radiation Medicine and Public Health, Soochow University, Suzhou, China; 2 Center for Radiological Research, College of Physicians & Surgeons, Columbia University, New York, NY, USA; 3 Department of Environmental Health Sciences, Mailman School of Public Health, Columbia University, New York, NY, USA; 4 Department of Radiation Oncology, College of Physicians and Surgeons, Columbia University, 630 West 168th St, New York, NY, 10032, USA

**Keywords:** TGFBI, Tumor suppressor, Mesothelioma, Breast tumor, Proliferation

## Abstract

**Background:**

Transforming growth factor β induced (TGFBI) product, an extracellular matrix (ECM) protein, has been implicated as a putative tumor suppressor in recent studies. Our previous findings revealed that expression of *TGFBI* gene is down-regulated in a variety of cancer cell lines and clinical tissue samples. In this study, ectopic expression of TGFBI was used to ascertain its role as a tumor suppressor and to determine the underlying mechanism of mesothelioma and breast cancer.

**Methods:**

Cells were stably transfected with pRc/CMV2-TGFBI and pRc/CMV2-empty vector with Lipofectamine Plus. Ectopic expression of TGFBI was quantified by using quantitative PCR and Western-blotting. Characterization of cell viability was assessed using growth curve, clonogenic survival and soft agar growth. The potential of tumor formation was evaluated by an *in vivo* mouse model. Cell cycle was analyzed via flow cytometry. Expressions of p21, p53, p16 and p14 were examined using Western-blotting. Senescent cells were sorted by using a Senescence β-Galactosidase Staining Kit. Telomerase activity was measured using quantitative telomerase detection kit.

**Results:**

In this study, an ectopic expression of TGFBI in two types of cancer cell lines, a mesothelioma cell line NCI-H28 and a breast cancer cell line MDA-MB-231 was found to have reduced the cellular growth, plating efficiency, and anchorage-independent growth. The tumorigenicity of these cancer cell lines as determined by subcutaneous inoculation in nude mice was similarly suppressed by TGFBI expression. Likewise, TGFBI expression reduced the proportion of S-phase while increased the proportion of G1 phase in these cells. The redistribution of cell cycle phase after re-expression of TGFBI was correspondent with transiently elevated expression of p21 and p53. The activities of senescence-associated β-galactosidase and telomerase were enhanced in TGFBI-transfected cells.

**Conclusion:**

Collectively, these results imply that TGFBI plays a suppressive role in the development of mesothelioma and breast cancer cells, possibly through inhibitions of cell proliferation, delaying of G1-S phase transition, and induction of senescence.

## Background

TGFBI, also called Betaig-h3, was first identified during the 1990s, when it was isolated from a human lung adenocarcinoma cell line (A549) which had been treated with TGF-β [1]. The TGFBI protein contains a secretary signal sequence (residues 1–23), four homologous internal domains, and a cell attachment (RGD) site [2,3]. TGFBI is secreted into the extracellular matrix (ECM) as an attachment protein. It functions mainly in cell adhesion, migration, proliferation, apoptosis, and angiogenesis [4-11]. Mutations of the *TGFBI* gene have been shown to be involved in several corneal dystrophies [12,13]. TGFBI mRNA and protein are up-regulated in different types of cell lines, including human epithelial cells, keratinocytes, lung fibroblasts, and melanoma cells. More recently, the *TGFBI* gene has been found to be frequently associated with cancer development. The expression of TGFBI is either down-regulated or lost in a variety of human tumor cell lines [4,14,15]. Transfection of TGFBI-expression plasmids into CHO cells led to a marked inhibition of tumor formation in nude mice. Ectopic expression of TGFBI in tumorigenic human bronchial epithelial cells induced by radiation and asbestos fibers significantly suppressed the tumorigenicity of those cells [3,14,16]. Recent findings have suggested that TGFBI also sensitizes ovarian cancer cells to paclitaxel by inducing microtubule stabilization and that the loss of TGFBI induces drug resistance and mitotic spindle abnormalities in ovarian cancer cells [17].

Malignant pleural mesothelioma (MPM) is an asbestos-related malignancy characterized by rapid, progressive, diffused growth and metastasis. The latency between tumor onset and the first exposure to asbestos or other carcinogenic fibers is extremely long, averaging over 30 years. Due to the long latency and extensive history of the use of asbestos in many industries, the incidence of MPM is expected to increase over the next few decades. It is estimated that about 2,500–3,000 new cases arise each year in United States and in Europe. An estimated 250,000 people will die of MPM in the next three decades [18,19]. Breast cancer, the most common malignancy in women living in western countries, has also been increasing in the rest of the world [20]. In the United States, breast cancer is the second most common cause of cancer deaths in women. Although the mechanism of how these two types of malignancy undergo malignant transformation remains largely unknown, evidence indicate a multistep process involving both activation of oncogenes and inactivation of tumor suppressor genes exists [21,22] The observation that many late-stage tumors are highly resistant to traditional chemotherapy and radiation therapy, highlights the need for innovative therapies based on mechanistic insight of the cancer process. In this regard, the potential role of TGFBI as a tumor suppressor may provide a novel target for manipulation and therapeutic purposes.

## Results

### Effects of TGFBI on tumor cell growth *in vitro*

**Figure 1:**
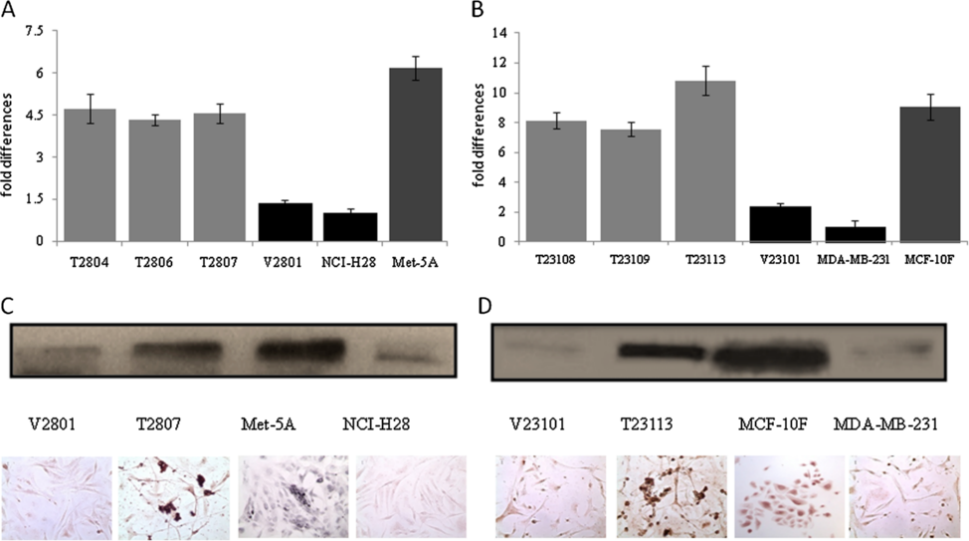
** Expression of TGFBI mRNA and protein in TGFBI-transfected, empty vector control, and parental tumor cells.** (**A**) Cells were transfected with pRc/CMV2-TGFBI and pRc/CMV2-empty vector. The expression of TGFBI mRNA was analyzed by quantitative real-time RT-PCR. The value was used to plot TGFBI expression using 2^▽▽Ct^. Each value is expressed in triplicate (error bars; mean ± SD). TGFBI-transfected mesothelioma cells (T2804, T2806, and T2807), pRc/CMV2- vector control cells (V2801), and parental mesothelioma cells NCI-H28. Met-5A is an immortalized human mesothelial epithelial cell line. (**B**) TGFBI-transfected breast cancer cells (T23108, T23109 and T23113), pRc/CMV2- vector control cell (V23101), and parental breast tumor cell MDA-MB-231. MCF-10 F is a spontaneously derived immortalized human breast epithelial cell line. Expression of TGFBI protein in (**C**) mesothelioma cells and (**D**) breast tumor cells was measured with Western blotting (upper) and immunohistochemical staining (lower, ×400).

**Figure 2:**
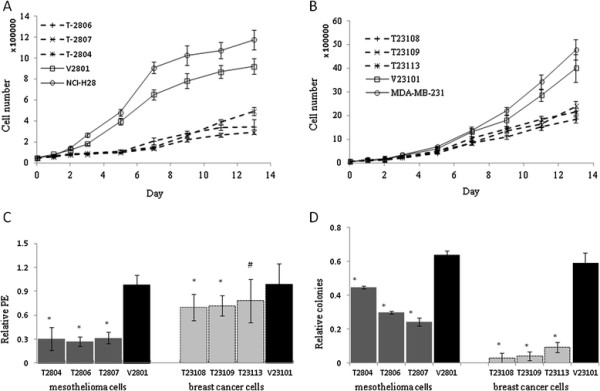
** Effects of TGFBI on the growth of tumor cells* in vitro*.** (**A**) Growth curves of TGFBI-transfected mesothelioma cells (T2804, T2806, and T2807), vector control cells (V2801), and parental mesothelioma NCI-H28 cells. (**B**) Growth curves of TGFBI-transfected breast cancer cells (T23108, T23109, and T23113), vector control cells (V23101), and parental breast tumor MDA-MB-231 cells. Data are generated from three independent experiments and error bars represent mean ± SD. (**C**) Relative plating efficiencies (PE) of TGFBI expression and vector control cells to parental cells, each in triplicate (error bars; mean ± SD). (**D**) Number of colonies expressing TGFBI or vector control relative to parental cells, each in triplicate (error bars; mean ± SD). * # indicates significant decreases relative to vector control cells (*, *P* < 0.01; #, *P* < 0.05).

Engineered mesothelioma cell clones (T2804, T2806, and T2807) and breast cancer cell clones (T23108, T23109, and T23113) ectopically expressing TGFBI were generated from their respective parental tumor cell lines, which only contained trace amounts of TGFBI. Representative clones were used for the study (Figures 1). To characterize the anti-proliferative and tumor suppressive effects of TGFBI, a growth kinetic study was conducted. The results demonstrated that the reintroduction of TGFBI into NCI-H28 and MDA-MB-231 cells dramatically slowed cell growth and prolonged population doubling time 4.38 and 1.16 times (Figures 2A and 2B), respectively. TGFBI also significantly reduced relative plating efficiency (PE), another parameter of cell viability. The plating efficiency of human mesothelioma cells dropped from 98.00% to 29.71%, and that of breast cancer cells dropped from 98.8% to 73.28% (Figures 2C). TGFBI expression inhibited anchorage-independent growth in these two cancer cell lines, exhibiting a drop of 48.54% in mesothelioma cells and 90.89% in breast cancer cells relative to control cells of both types (Figures 2D). These results suggest that TGFBI modulates cell proliferation and neoplastic transformation phenotypes.

### Effects of TGFBI on tumor development *in vivo*

To determine whether TGFBI has a tumor-suppressive effect *in vivo*, we subcutaneously inoculated TGFBI-expressing tumor cells and vector control cells (herein referred as controls) into immuno-deficient nude mice. Tumor formation was monitored by weekly palpation and by direct nodule resection. We found that tumor nodules were palpable as early as 4 weeks after inoculation in the mice injected with vector control breast cancer cells. By 5 weeks, 100% of the mice grew tumors, with an average volume of 448 mm^3^. In contrast, mice injected with TGFBI-expressing cells (T23108, T23109, and T23113) showed signs of tumor growth at 6 weeks post inoculation, 2 weeks later than control groups. Only 50% of these mice developed tumors by 12 weeks (Table 1), and the average tumor volume was only 252 mm^3^ (#, *P* < 0.01).

**Table 1:** **Suppression of *in vivo *tumor growth by ectopic expression of TGFBI in breast cancer cells**

	**palpable nodules/point of injection**		**tumor volume at 12 w (mm^3^)**	
MDA-MB-231	12/12	}24/24	452.67 ± 114.56	}448.36 ± 107.56
V23113	12/12		444.05 ± 105	
T23108	4/8	}12/24*	o ± 41.88	}251.85 ± 36.16^#^
T23109	5/8		264.55 ± 28.94	
T23113	3/8		225 ± 32.69	

*, Chi-squared text, *P* < 0.01, relative to parental and empty vector tumor cells

#, t-test, *P* < 0.01, relative to parental and empty vector tumor cells.

**Figure 3:**
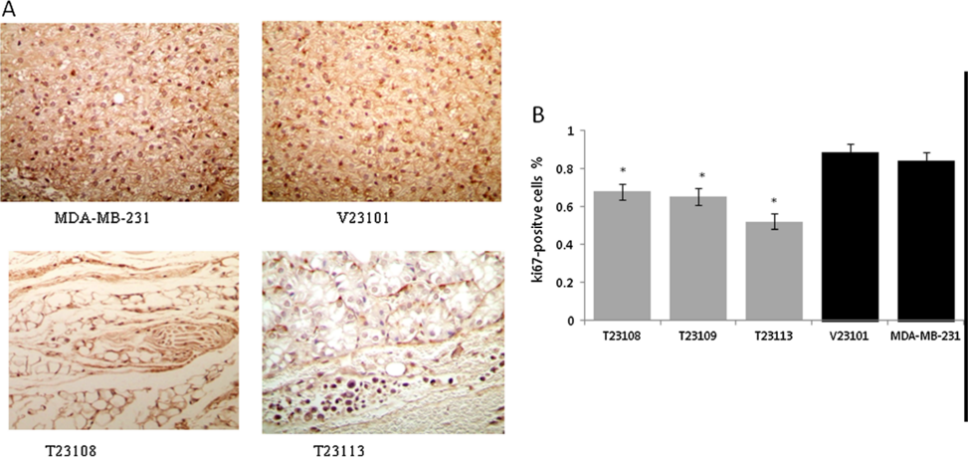
** Effects of TGFBI on cell proliferation* in vivo*.** Expression of ki67 in immuno-deficient nude mice subcutaneously injected with MDA-MB-231 cells transfected with TGFBI or vector control was assayed using immunohistochemical staining. (**A**) More brown than blue nuclei were observed in ki67-positive cells. (**B**) Data are shown as the number of ki67 positive cells relative to V23101 cells, *, *P* < 0.01.

In order to show the inhibitory effects of TGFBI on tumor growth at the molecular level, ki67, a molecular marker of cell proliferative capacity was used to stain the tissue slides dissected from tumors of each group [23]. Our results showed that there were significantly fewer ki67-positive cells in tumor tissues expressing TGFBI than in tissues without TGFBI (Figures 3). This supports the assertion that TGFBI inhibits cell proliferation *in vivo*.

### Effects of TGFBI on G1 phase arrest and S phase delay

**Figure 4:**
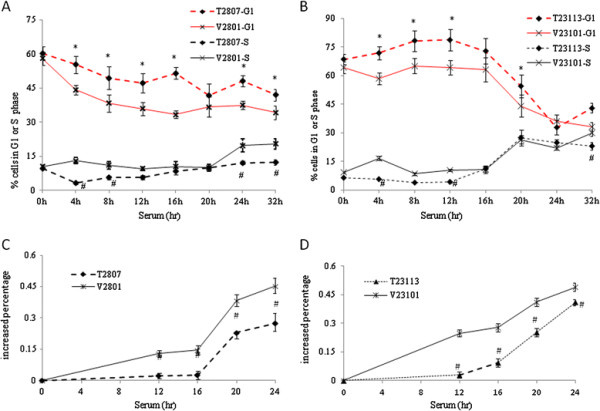
** TGFBI during G1 phase arrest and S phase delay.** (**A**) Mesothelioma and (**B**) breast tumor cell lines with and without re-expressed TGFBI were collected at different times, cell cycle profiles were assessed by flow cytometry. Line graphs and plots illustrate the distribution of cells in G1 and S phases over 32 h. Representative proliferation of (**C**) mesothelioma and (**D**) breast tumor cell lines with and without TGFBI re-expression assessed using a CyQUANT NF proliferation kit at the indicated points in time. The proliferation rate is expressed as increased percentages [(fluorescence intensity at time t-fluorescence intensity at 0 h)/fluorescence intensity at 0 h]. All growth data were generated from three independent experiments (error bars; mean ± SD). * Indicates significant increases over vector control cells (*P* < 0.05). # Indicates significant decreases relative to vector control cells (*P* < 0.05).

To determine whether the suppressive effects of TGFBI on cell proliferation and subsequent transformation were due to alterations in cell cycle progression, we compared cell cycle profiles between TGFBI-transfected and control cells in these two types of tumor cell lines. After serum starvation, both control and TGFBI-expressing cells were largely arrested in G1 phase, as shown in Figures 4A and 4B. With serum stimulation, the proportion of cells in the G1 phase was far lower in control cells than in TGFBI-expressing cells. (*, *P* < 0.05). These control cells began to enter the S phase as early as 4 h after serum stimulation, but TGFBI-expressing cells did not begin to enter the S phase before 20 h. Although the number of TGFBI-expressing mesothelioma cells in the S phase increased over time, it remained significantly lower than that of the control cells at all evaluated points in time, specifically 4, 8, 24, and 32 h after serum stimulation. Similar changes were observed in breast cancer cells (#, *P* < 0.05). When TGFBI was expressed, the cell proliferation rate (T2807 and T2313) was lower than that of control cells at 12–24 h after serum stimulation (#, *P* < 0.05; Figures 4C and 4D). These results imply that TGFBI-expressing cells may be more resistant to cell cycle transition (G1-S) than other cells, even when exposed to external stimulation.

**Figure 5:**
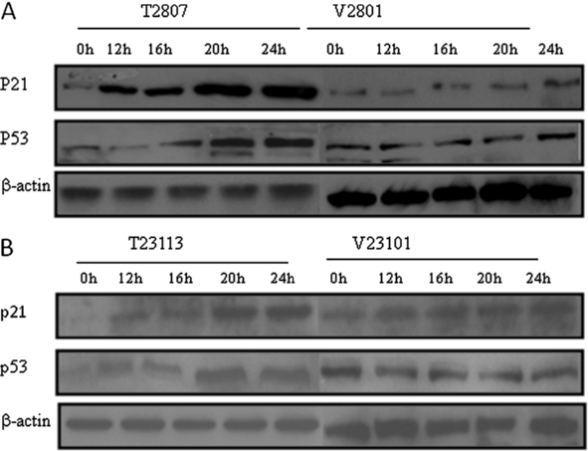
** TGFBI up-regulates expression of p53 and p21.** (**A**) Mesothelioma and (**B**) breast tumor cell lines transfected with vector and TGFBI were synchronized in quiescence by serum starvation and induced to reenter the cell cycle by the addition of serum. Temporal expression of p53 and p21 in response to serum stimulation were assessed by Western blotting.

Tumor suppressors p53 and p21 are known to regulate the G1/S checkpoint. Their expression levels were therefore examined in TGFBI-expressing cells and in controls, as shown in Figures 5A and 5B. TGFBI-expressing cells T2807 and T23113 exhibited elevated p21 and p53 levels at 12 h and up to 24 h upon serum stimulation. In contrast, their expression in control cells showed less significant changes, indicating that TGFBI delayed S-phase entry and that this phenomenon may be associated with the up-regulation of p21 and p53.

### Effects of TGFBI on cellular senescence

**Figure 6:**
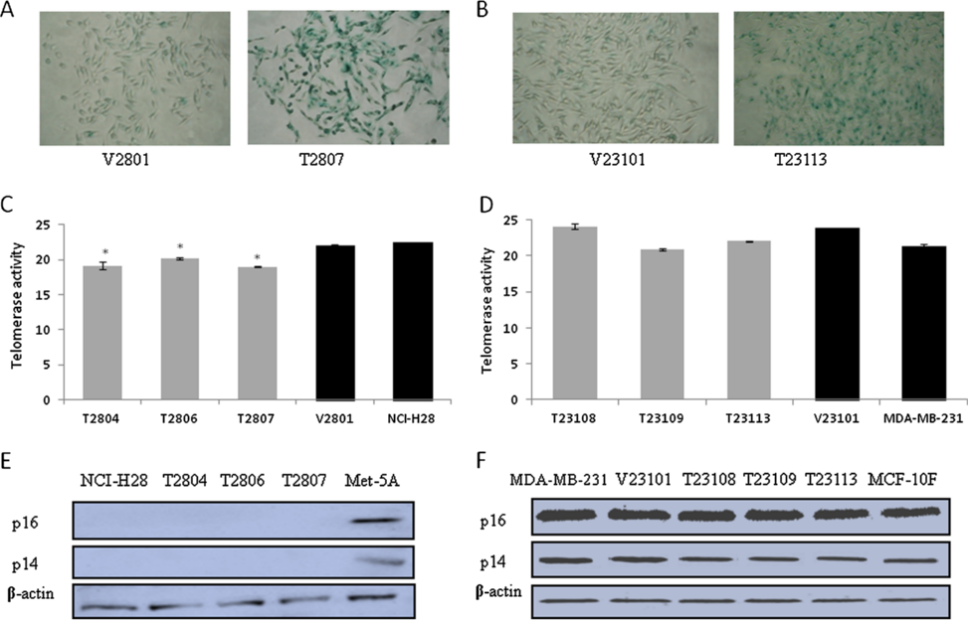
** TGFBI induces cellular senescence.** (**A**) Mesothelioma and (**B**) breast cancer cell lines with and without re-expression of TGFBI were sub-cultured and then maintained for 2 days in growth medium prior to assay for SA-β-Gal staining. Most of the T2807 and T2313 cells were SA-β–Gal-positive (right panels of A, B, ×200), and most of the V2801 and V23113 cells were SA-β–Gal-negative (left panels of A, B, ×200). (**C**) Mesothelioma and (**D**) breast tumor cell extracts were prepared, and telomerase activity was assayed using QTD real-time PCR. Data are shown as the threshold cycle (Ct) of real-time PCR relative to vector control cells, *, *P* < 0.01. Expression of p16 and p14 in (**E**) mesothelioma and (**F**) breast tumor cells was assessed by Western blotting with whole-cell lysates from harvested cells.

Senescence is an aging state during which cells lose the ability to divide, which is often controlled by some oncogenes and tumor suppressors [24-26]. Dysregulation of senescence can lead to cellular immortalization and malignant transformation. Senescence-associated β-galactosidase (SA-β-Gal) has frequently been used as a marker of cellular senescence, as indicated by histochemical staining at pH 6.0 [27]. In this study, strong positive staining was observed for β-galactosidase activity in most of TGFBI-expressing cells, such as T2807 and T23113, but not in control cells (Figures 6A and 6B). These results suggest that TGFBI may be involved in the regulation of cellular senescence. One of the proposed scenarios is that cells are “pushed” into senescence by telomere shortening, which is facilitated by telomerase activity. Immortalized cell lines and/or tumor cells gain the ability to maintaining their telomeres through alternative lengthening mechanisms [28]. Our data show that the telomerase activity of TGFBI-expressing mesothelioma cells is significantly higher than that of controls (*P* < 0.01, Figures 6C). This is consistent with TGFBI’s hypothesized inhibitory role. However, two well-known senescence regulators, p16 and p14, were found to be unaffected by TGFBI re-expression (Figures 6E). This indicated that TGFBI could not recovery expression of p16 and p14 in mesothelioma cells with biallelic deletion. In breast cancer cells, neither the telomerase activity (Figures 6D) nor expression of p16 and p14 (Figures 6F) changed in response to re-expression of TGFBI.

## Discussion

TGFBI, an extracellular secreted matrix protein, was originally implicated as a regulator of cell adhesion and migration. More recently, down-regulation of TGFBI expression has been reported to be involved in the development of human tumors, including lung, breast, ovarian, prostate, embryonic rhabdomyosarcoma, insulinoma, and mesenchymal tumors [14,16,29-33]. Loss of TGFBI expression has also been observed in neoplastic transformation in CHO cells and papillomavirus-immortalized human bronchial epithelial cells [3,14,16].

The physiological role of TGFBI is still largely unknown. It has been reported that the embryonic expression of TGFBI is particularly strong in the mesenchyme of many tissues throughout all stages of development [34]. In addition, immunohistochemical analysis has demonstrated that TGFBI proteins are deposited in ECM and in cytoplasm and nuclei. Analyses of medium and matrix fractions displayed a protein at 70–74 kDa, and nuclear extracts showed a 65 kDa reactive protein band [35]. We also found that TGFBI protein localized not only in cell culture medium and cytoplasm, but also in the nuclei of TGFBI-transfected tumor cells and immortalized epithelial cells (Met-5A cells and MCF-10 F cells). The diverse distribution of TGFBI suggests that the functions of TGFBI may not be limited to its role as a component of ECM.

The FAS1 domains of TGFBI have been shown to inhibit tumor angiogenesis and tumor growth and to promote apoptosis. This is also consistent with a tumor suppressor role for TGFBI [36]. Recent evidence has shown that TGFBI expression causes significantly higher sensitivity to apoptotic induction by upregulation of IGFBP3 [29]. It also repressed tumor cell invasion, possibly by suppressing the PI3K/Akt/mTOR signaling pathway [37]. Loss of TGFBI expression is frequent in human cancer and it has been causally related to acquisition of tumorigenic phenotype in asbestos-treated immortalized human bronchial epithelial cells. In this study, by re-introduction of TGFBI into tumor cell lines MDA-MB-231 and NCI-H28, which have naturally low levels of TGFBI, we substantiated the role of TGFBI as a tumor suppressor and more importantly discovered previously unknown portions of its underlying mechanism.

Our data show that TGFBI significantly reduced cell growth rate, plating efficiency, and anchorage-independent growth. These parameters are often used to assess the fundamental characteristics linked to the functions of oncogenes and tumor suppressors. The results are consistent with proposed biological functions of TGFBI and results obtained from this and previous studies [1,36]. Cell cycle progression through G1 phase into S phase is a major checkpoint for cells during proliferation. Dysregulation of the G1/S transition may arrest the cells in quiescence or drive them into nonstop proliferation, depending on the specific scenario. A number of oncogenes and tumor suppressors affect the G1/S transition directly or indirectly, notably cyclin A1, p21, and p53 [38]. Data from this study demonstrates that TGFBI upregulates p53 and p21. This suggests that the inhibitory effect of TGFBI on this checkpoint may be related to these two molecules.

Earlier, our group presented evidence that TGFBI deficiency can lead to mutations, chromosomal fragmentation, and genetic instability, which in turn promotes tumor development. Similarly, ablation of TGFBI increases the frequency of chromosomal aberration and micronuclear formation, as observed in fibroblast cells isolated from TGFBI knock-out mice. However, these cells also showed more proliferation and earlier entry into S-phase entry than those of wild-type mice [39]. In this study we did not check for genetic instability but rather precisely reproduced the evidence of TGFBI’s inhibitory effects on cell proliferation, transformation, and G1/S transition using a different model, which strongly supported the conclusion that TGFBI is a tumor suppressor. It may execute its function by modulating or interacting with other cell cycle effectors, ultimately leading to unchecked cell proliferation and malignant transformation.

Cellular senescence is defined as a state of irreversible arrest in cell division after a period of serial proliferation in normal diploid cells [40]. It can also serve as a stress protective response. It can be triggered by a number of sensing mechanisms, such as telomere shortening, epigenetic derepression of the INK4a/ARF locus that encodes two physically linked tumor suppressor proteins p16/p14, and DNA damage [41]. p16 has been shown to inhibit the ability of cyclin D1 to hinder S-phase entry, which is one of the possible mechanisms involved in the regulation of cellular senescence [42]. Escaping senescence is a prerequisite for cell immortalization and transformation [43-45]. We therefore asked if TGFBI’s inhibitory effect on cellular transformation (anchorage-independent growth and malignancy) is related to its modulation of senescence. To our surprise, an enhanced senescence accompanied by the expression of TGFBI was evidenced by the increased levels of SA-β-gal, a classic marker of cellular senescence.

Elevation of telomerase activity, another sign of senescence, however, exhibited different pattern in mesothelioma and breast cancer cells. In NCI-H28 cells, telomerase activity increased significantly with the expression of TGFBI, which directs cells into senescence. The loss of TGFBI is therefore believed to contribute to the escape of cells from senescence. However, TGFBI did not affect telomerase activity in MDA-MB-231 cells. The expression of p16 and p14 showed no significant difference between TGFBI-expressing and control cells. Homozygous deletion of the p16 gene has been reported in 85% of mesothelioma cell lines, including NCI-H28 cells and 22% of primary tumor specimens [46,47]. This makes it difficult to assess the functional association between TGFBI and p16. Other mechanisms may be involved in controlling the process, p21 and p53 are potential candidates [48,49]. In both types of cells, p21 and p53 were both up-regulated upon TGFBI expression. Our results clearly showed that SA-β-gal and telomerase activity were both up-regulated by TGFBI re-expression. This may suggest that TGFBI carries out its inhibitory functions on cellular senescence involving p21 and p53.

Further results derived from *in vivo* substantiated the role of TGFBI as a tumor suppressor. After implanting cells with TGFBI and leaving others without, we analyzed the onset, incidence, and volume of the resulting tumors in mice, in order to assess the tumor suppressive effect of TGFBI. Although TGFBI did not completely block the formation of tumors derived from injection of MDA-MB-231 cells, the onset of tumor formation was delayed, tumor volume was greatly reduced, and the number of tumors decreased dramatically. This is in accordance with our previous data, which showed that TGFBI suppresses tumorigenic phenotypes in lung and human bronchial epithelial cells induced by radiation and asbestos [14,16,29]. Unfortunately, both TGFBI-expressing and vector control meosthelioma cells failed to produce progressively growing tumors even at 5 months after cell inoculation. We are not sure what the exact reason for this may be. One explanation could be that the residual immunity of nude mice may still be able to reject some types of cells. One alternative means of evaluating these phenomena would be to use SCID mice, which lack both T and B lymphocytes, unlike nude mice, which only lack T cells. For further evaluation of the inhibitory role of TGFBI on a molecular level, tissue slides dissected from tumors in each group were stained with the nuclear antigen ki67, which serves as a marker of cellular proliferation capacity [23,50]. The number of ki67-positive cells inversely correlated with the level of TGFBI expression; the more ki67-positive cells observed in the vector control groups, the stronger the evidence that TGFBI diminishes the ability of cells to proliferate and therefore inhibits tumorigenicity *in vivo*. We here present strong evidence that unequivocally supports that TGFBI exhibits an inhibitory effect on tumor growth both *in vitro* and *in vivo*, especially in mesothelioma and breast cancer cells.

Contrary evidence from other groups was brought into our attention. For example, it has been suggested that TGFBI increases the metastatic ability of colon and an ovarian cancer cell lines [51,52] In addition, TGFBI has been shown to be over-expressed in pancreatic cancer, renal cell carcinoma and glioblastoma [53-55]. It is likely that TGFBI protein may function in multiple ways depending on tissue type and tumor microenvironment. *TGFBI* gene is a downstream target of transforming growth factor beta (TGF-β) that inhibits the proliferating of normal epithelial cells and functions as a tumor suppressor in early tumorigenesis as well as a tumor promoter in later stage of tumor progression. This stage-specific dual functional role of TGFBI in cancer represents an emerging paradigm whereas the mechanism behind is not well understood. We are planning to expand our research to more type of cell lines and clinical samples. Our particular focus will be on the questions left unanswered by this and other reports.

## Conclusion

In summary, our study is the first to show that TGFBI inhibits cell proliferation and transformation by delaying G1-S phase transition and inducing cellular senescence in mesothelioma and breast cancer cells, indicating that TGFBI may serve as a negative regulatory effector and potential tumor suppressor in the development of malignances such as mesothelioma and breast cancer. Our findings may offer a new vision for the management of certain types of cancers.

## Methods

### Cell culture and stable transfection of TGFBI

Human malignant pleural mesothelioma cell line (NCI-H28) and a breast tumor cell line (MDA-MB-231) were obtained from the American Type Culture Collection (ATCC; Manassas, VA, U.S.) and grown in Dulbecco’s Modified Eagle medium (DMEM) supplemented with 10% fetal bovine serum (FBS). Cells were plated into 6-well plates and transfected with either pRc/CMV2-TGFBI or pRc/CMV2-empty vector with Lipofectamine Plus (Invitrogen, Carlsbad, CA, U.S.). The cells were split at 1:10 and cultured in medium containing 700 μg/ml of G418 (Sigma-Aldrich, St. Louis, MO, U.S.) for 21 d**.** Resistant colonies were isolated, expanded in cultures, and maintained in the presence of 300 μg/ml of G418.

The expression of TGFBI mRNA was analyzed by quantitative real-time reverse transcription-PCR (RT-PCR; Applied Biosystems 7300, Foster City, CA, U.S.) using a RT^2^ Real-time SYBR Green/ROX Gene Expression Assay Kit (SuperArray Bioscience Corp., Frederick, MD, U.S.). The first strand of cDNA was synthesized from 4 μg total RNA using SuperScript II First-Strand Synthesis System (Invitrogen). Relative quantification of TGFBI mRNA expression was performed using real-time PCR. A comparative threshold cycle (Ct) was used to determine the expression level. The expression levels of TGFBI mRNA were expressed as an n-fold difference relative to the calibrator. Briefly, the TGFBI mRNA Ct value was normalized using the following formula: ▵Ct = Ct_*TGFBI*_ - Ct_*GAPDH*_. To determine relative expression levels, the following formula was used: ▵▵Ct = ▵Ct_*sample*_ - ▵Ct_*calibrator*_. The resulting values were used to plot the TGFBI expression using the expression 2^▵▵Ct^.

Expression of the TGFBI protein in the supernatant of cells was confirmed by Western blotting. Cells were plated and grown in DMEM with 10% FBS for 24 h. They were then transferred to serum-free medium and maintained for another 24 h. The medium was then harvested and trichloroacetic acid (TCA) was added to a final concentration of 10%. It was then incubated at RT for 30 min, centrifuged with 13,000 rpm at 4°C for 30 min, and the supernatant was aspirated. The pellet was washed three times with acetone and then air dried. Fifty microliters of laemmli sample buffer was added to the pellet and boiled for 5 min. It was then resolved on SDS-PAGE. The gels were transferred onto PVDF membrane and incubated serially with monoclonal anti-human TGFBI (R&D Systems, Minneapolis, MN, U.S.) followed by sheep anti-mouse IgG conjugated with horseradish peroxidase as secondary antibody (Amersham Biosciences, Piscataway, NJ, U.S.). Multiple clones were chosen for the study, and similar results were observed with each. The results shown in this manuscript are representatives of the findings.

### Immunohistochemical staining

The expression of TGFBI and Ki-67 was measured by immunohistochemical staining. Cells were fixed in 4% paraformaldehyde and then incubated in 0.3% hydrogen peroxide in absolute methanol for 30 min to quench the endogenous peroxide activity. Immunostaining was performed with a Vestastain Elite ABC Kit (Vector Laboratories, Burlingame, CA, U.S.). Briefly, the slides were blocked with horse serum for 30 min and then incubated with anti-human TGFBI antibody or anti-mouse Ki-67 antibody (Santa Cruz Biotechnology, CA, U.S.) overnight at 4°C. After washing with PBS, biotin-conjugated secondary antibody was applied to the slides for 30 min, followed by avidin-biotin-peroxidase complex for 30 min. The slides were then exposed to a reaction solution containing the chromogen, 3,3′-diaminobenzidine (DAB) for 6 min, washed with distilled water, and counterstained with Meyer’s hematoxylin for 10 s. The slides were dehydrated, cleared, and mounted. The slides were examined and representative pictures were captured using an Olympus B × 60 camera. More brown nuclei than blue were noted for ki67-positive cells. Five hundred cells on each slide were evaluated using 40× magnification over the hotspot. Data are shown as number of ki67 positive cells relative to the number of V23101 cells, *, *P* < 0.01.

### Growth curve assay

Five thousand cells were plated in 35 mm dishes in complete medium. The medium was changed every 3 days. At specific points in time after plating (Days 0, 1, 2, 3, 5, 7, 11, and 13), cells were trypsinized and the number of cells was determined using a Coulter Counter (Beckman Coulter Inc. Miami, FL, U.S.). The doubling time of the culture was analyzed using the formula: Nt = N_0_ 2^tf^; doubling time = 1/f; Nt: number of cells at time t; N0: initial number of cells; t: time (days); f: frequency of cell cycles per unit time.

### Clonogenic survival assay

Cells were trypsinized and counted with a Coulter Counter. Aliquots of the cells were seeded into dishes 100 mm in diameter. After two weeks of incubation at 37°C and 5% CO_2_, the colonies formed were fixed with formaldehyde, stained with Giemsa, and counted using an Oxford Optronix Colony Counter (Oxford Optronix Company, UK). The relative plating efficiencies (PE) were determined using the following formula: Relative PE = number of colonies of TGFBI expression or vector control cells / number of colonies of parental cells.

### Soft agar assay

Two thousand cells were mixed with 1 mL of 0.35% agarose and plated into 35 mm dishes with a bottom layer of 0.75% agarose. Cells were fed every 3 days with 1 ml culture medium. The colonies were counted two weeks after initial plating. Data are presented as ratio of number of colonies of TGFBI expression or vector control cells / number of colonies of parental cells. Data points in figures represent three independent experiments.

### Cell cycle analysis

Cells were arrested in quiescence by serum starvation in serum-free DMEM medium supplemented with 1% bovine serum albumin (BSA) for 36 h. Cells were stimulated to reenter the cell cycle by replenishing with fresh medium containing 10% serum. At different points in time after serum stimulation, cells were fixed with ice cold 75% ethanol. Cells were labeled with propidium iodide (PI) and analyzed using a FACSCalibur flow cytometer (BD Biosciences, San Jose, CA, U.S.). Line graphs and plots illustrate the distribution of cells in the G1 and S phases over a period of 32 h. Comparisons between TGFBI-transfected cells and empty control cells were determined using the Student’s t-test. *, *P* <0.05 was considered to be significant.

### Cell proliferation assay

Proliferation was assessed by CyQUANT NF Cell Proliferation Assay according to the manufacturer’s instructions (Invitrogen, Molecular Probes, Inc. Eugene, OR, U.S.). Briefly, after 0, 12, 16, 20, and 24 h of serum stimulation, cells were washed with PBS and incubated for 1 h with the fluorescence substrate. Fluorescence intensity was measured on an automatic microplate reader (Bio-Tec, Winooski, VT, U.S.). Data are shown as in percentages: [(fluorescence intensity at time t – fluorescence intensity at 0 h) / fluorescence intensity at 0 h] * 100%.

### Western blotting

Proteins were extracted with lysis buffer (50 mM Tris–HCl, pH 8.0, 150 mM NaCl, 1% NP-40, 0.1% sodium dodecyl sulfate, 1 mM phenylmethylsulfonyl fluoride and protease inhibitor cocktails). Protein concentrations were determined using the Bio-Rad protein Assay (Bio-Rad, Hercules, CA, U.S.). Equal amounts of protein (30 μg) were fractionated by SDS-PAGE and transferred onto PVDF membranes under semi-dry conditions. Antibodies against p21, p53, p16, p14, and β-actin were obtained from Cell Signaling Technology, Inc. (Danvers, MA, U.S.). Secondary antibodies were purchased from Amersham Biosciences and signals were detected using an enhanced chemiluminescence (ECL) method according to the manufacturer’s instructions (Amersham Biosciences, Piscataway, NJ, U.S.).

### Senescence associated β-galactosidase staining

Senescent cells were detected using a Senescence β-Galactosidase Staining Kit (Cell Signaling Technology Inc, MA, U.S.). Briefly, cell monolayers were washed twice with PBS and then fixed with fixative solution for 15 min. The cells were then washed twice with PBS. Staining solution (930 μl Staining Solution; 10 μl Staining Supplement A; 10 μl Staining Supplement B; 50 μl 20 mg/ml X-gal in DMF) was applied and then the cells were incubated at 37°C for 16 h. After incubation, the cells were washed twice with PBS and photographed using an Olympus camera.

### Telomerase activity

Telomerase activity was evaluated using quantitative telomerase detection kit (QTD kit, US Biomax, Inc, MD, U.S.). Briefly, cells were lysed in 1 × lysis buffer and incubated at 4°C for 30 min. The lysate was then centrifuged at 12,000 × g for 20 min at 4°C, and the supernatant was collected. The protein concentration of the cell lysate was determined using a Bio-Rad protein Assay. Standards, inactivated samples, and template-free reactions were also assayed on every plate for quality control purposes. Each sample was analyzed in triplicate. Real-time amplifications were performed on an ABI Prism 7300 Sequence Detection System (Applied Biosystems, CA, U.S.). A comparative threshold cycle (Ct) was used to determine telomerase activity, which is negatively related to the Ct of real-time PCR.

### Tumorigenicity *in vivo*

Male Nu/Nu mice (purchased from Harlan Sprague–Dawley, Inc. Indianapolis, IN, U.S.) were housed under pathogen-free conditions. The animals were lightly anesthetized with isoflurane, and then 5×10^6^ of parental, vector control, and TGFBI-transfected tumor cells were injected subcutaneously into the left and right flanks of each animal (Anaquest, Madison, WI, U.S.). Animals were maintained under sterile conditions for 5 months and palpated weekly for tumor formation. Animals were killed as soon as tumor nodules reached a size of 0.5–0.8 cm. All animal studies were conducted at Columbia University under strict Institutional Animal Care and Use Committee-approved protocols.

### Statistical analysis

Data are presented as mean ± standard deviation (SD). Data were subjected to one way analysis of variance (ANOVA) and comparisons between TGFBI-transfected cells and empty vector control cells were determined using the Student’s t-test. Differences were considered statistically significant at *P* <0.05.

## Competing interests

The authors declare that they have no competing interests.

## Authors’ contributions

Bingyan Li, Gengyun Wen, and Yongliang Zhao performed the experiments, Bingyan Li provided data analysis, Bingyan Li, Gengyun Wen, Jian Tong, and T. K. Hei designed the study and participated in writing the paper. All authors read and approved the manuscript.

## Pre-publication history

The pre-publication history for this paper can be accessed here:

http://www.biomedcentral.com/1471-2407/12/239/prepub
